# Different Types of Errors in Saccadic Task Are Sensitive to Either Time of Day or Chronic Sleep Restriction

**DOI:** 10.1371/journal.pone.0126502

**Published:** 2015-05-26

**Authors:** Barbara Wachowicz, Ewa Beldzik, Aleksandra Domagalik, Magdalena Fafrowicz, Magda Gawlowska, Justyna Janik, Koryna Lewandowska, Halszka Oginska, Tadeusz Marek

**Affiliations:** 1 Department of Cognitive Neuroscience and Neuroergonomics, Institute of Applied Psychology, Jagiellonian University, Krakow, Poland; 2 Neurobiology Department, Malopolska Centre of Biotechnology, Jagiellonian University, Krakow, Poland; 3 Institute of Culture, Jagiellonian University, Krakow, Poland; University of Muenster, GERMANY

## Abstract

Circadian rhythms and restricted sleep length affect cognitive functions and, consequently, the performance of day to day activities. To date, no more than a few studies have explored the consequences of these factors on oculomotor behaviour. We have implemented a spatial cuing paradigm in an eye tracking experiment conducted four times of the day after one week of rested wakefulness and after one week of chronic partial sleep restriction. Our aim was to verify whether these conditions affect the number of a variety of saccadic task errors. Interestingly, we found that failures in response selection, i.e. premature responses and direction errors, were prone to time of day variations, whereas failures in response execution, i.e. omissions and commissions, were considerably affected by sleep deprivation. The former can be linked to the cue facilitation mechanism, while the latter to wake state instability and the diminished ability of top-down inhibition. Together, these results may be interpreted in terms of distinctive sensitivity of orienting and alerting systems to fatigue. Saccadic eye movements proved to be a novel and effective measure with which to study the susceptibility of attentional systems to time factors, thus, this approach is recommended for future research.

## Introduction

Throughout the day, cognitive performance stays under the combined influence of homeostatic sleep pressure and circadian processes. Interplay between these two factors affects the performance of everyday tasks and interferes with a contemporary lifestyle characterized by a large accumulation of duties which require continuous and prolonged cognitive efficiency. Such demands result in a reduction in sleep time, which has become a hallmark of modern society. Yet, most laboratory experiments deal with total sleep deprivation assuming that the consequences of chronic partial sleep restriction are comparable to those of total sleep loss [1]. The effects of sleep restriction are a general decrease in alertness, wake state instability (including lapses of attention and microsleeps), perseveration of ineffective solutions, and weakened memory/learning performance [2]. However, sustained attention deteriorates much more than the performance of challenging working memory tasks [3].

Even in a state of rested wakefulness, circadian rhythm driven by endogenous (eg. the circadian process and sleep pressure resulting in sleepiness and fatigue) as well as exogenous factors (eg. lighting and social environment) lead to variations in cognitive functioning [4]. However, assessing human cognitive performance rhythms is complicated by two masking factors: the kind of task used and inter-individual differences in task performance [5]. Moreover, the multidimensional nature of attention itself is probably the source of inconsistencies between the various studies of the circadian and homeostatic effects on performance relevant to the attentional domain investigated [4]

Errors arising due to sleep pressure and/or circadian rhythm may be related to either response selection or execution process. Their characteristics would vary depending on the neuronal network involved: alerting, orienting or executive control [6], which are responsible for maintaining optimal vigilance, ordering sensory input and top-down regulation, respectively. Errors may also be differentiated depending on sleep pressure and the circadian process. As suggested by Valdez and colleagues [7] vigilance (sustained attention, concentration) is linked to fatigue, i.e. the homeostatic process, while tonic alertness (general capacity to respond), phasic alertness (the capacity to respond to a stimulus after a warning signal) and selective attention (the capacity to respond to a specific stimulus and ignore others) show circadian variations.

Research on sleep deficit in terms of alertness and vigilance has concentrated mostly on manual responses [8]. The few experiments that have addressed oculomotor responses, have focused mostly on the velocity and latency of eye movements (eg. [9]). The typology of the saccadic reactions is little explored—especially in the context of chronic sleep loss or circadian rhythms. However, saccadic responses provide a valid model for investigating basic cognitive processes, especially attentional capacities, and flexible control over behaviour. They are controlled by different low- and high-level systems [10] and by separate excitatory and inhibitory neural pathways [11]. These neural pathways are strictly linked with the top-down and bottom-up attentional processes, influencing eg. cue facilitation mechanisms in saccadic eye movement [12]. Thus, different types of saccadic task errors should be considered failures of diverse attentional functions.

The aim of this study was to verify the effects of chronic sleep deficit and time of day on human saccadic task performance. We hypothesized that those conditions would differentiate the oculomotor behaviour, and, in consequence, provide new information about the functioning of attentional sub-systems.

## Materials and Methods

### Participants

Twenty four paid volunteers participated in this experiment (12 females, mean age 22.7 ± 1.6 years). Participants met the experiment requirements: right-handedness, right-eyed, normal or corrected-to-normal vision, no physical or psychiatric disorders. They were all non-smokers and drug-free. The absence of sleep problems or excessive sleepiness was confirmed by the Pittsburgh Sleep Quality Index [13], and the Epworth Sleepiness Scale [14]. Mean sleep quality index was 4.08 (std. err. 0.46) and the daytime sleepiness score was 5.71 (std. err. 0.53). Neither extremely morning- nor evening-oriented subjects were qualified for the study (Chronotype Questionnaire; [15]).

### Ethics Statement

Participants were informed about the procedure and goals of the study and gave their written consent. The study was approved by the Bioethics Commission at the Jagiellonian University.

### Procedure

The experimental task was performed in two conditions: rested wakefulness (RW—after a week with unrestricted, fully restorative sleep) and chronic sleep deficit (SD, after seven days of sleep curtailment). The order of experimental sessions (RW and SD) was counterbalanced across all participants. The sessions were separated by at least two weeks in order to minimize the residual effects of sleep deficit on performance. In the SD condition, the participants were instructed to shorten their sleep by delaying sleep time and using an alarm clock in the morning. The sleep length was individually calculated for them as two thirds of the self reported optimal sleep duration. The actual sleep duration and the sleep timing were verified on the basis of movement recorded by a Micro Motionlogger SleepWatch (Ambulatory Monitoring, Inc., Ardsley, NY) which was worn during the week before each experimental day on the participant’s non-dominant wrist. According to actigraphy measurements, the sleep length in the SD condition was reduced by 30% in comparison to the RW condition. The severity of subjective sleep loss consequences was assessed with a CHICa scale [16].

All the participants performed an experimental task four times during the day: at approximately 10:00, 14:00, 18:00 and 22:00. Before each measurement session, participants were asked to estimate their alertness using the Karolinska Sleepiness Scale (KSS; [17]). A semi constant routine protocol was applied: room temperature and light intensity were kept constant, caloric intake and the level of motor activity were controlled. The participants spent approximately 14 hours in a controlled laboratory environment. During experimental days, they were allowed to engage in non-strenuous activities (eg. reading, watching videos, conversation). Caffeine intake was banned; alcohol consumption during the preceding week was restricted.

### Stimuli and task

**Fig 1:**
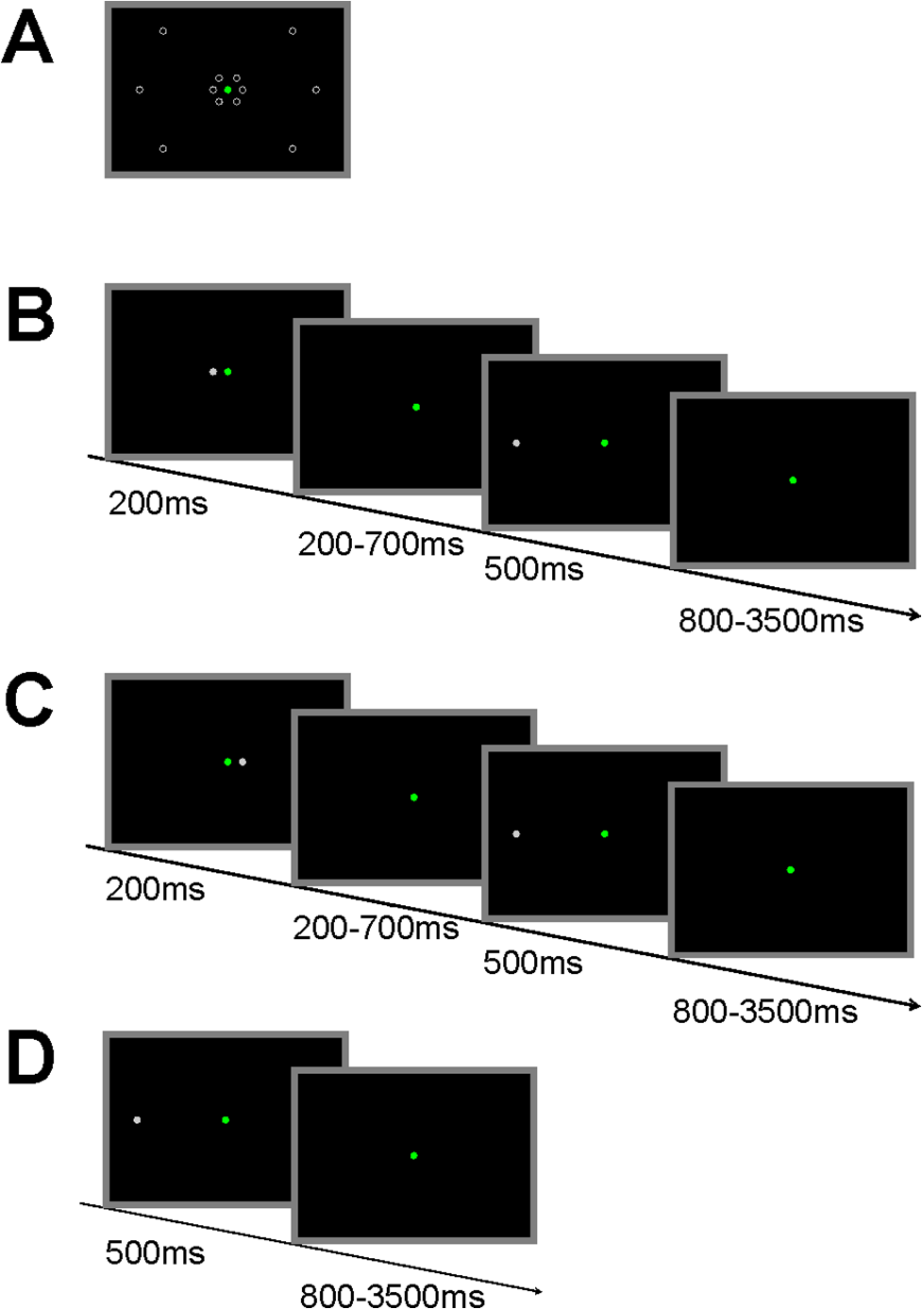
Experimental task used in the study. (A) Possible locations for cues and targets; (B) Congruant trial; (C) Incongruent trial; (D) Trial without a cue.

A modified spatial cueing task [18] was prepared using E-Prime 2.0 (Psychology Software Tools) and presented on a 17-inch screen located approximately 80cm from participants’ eyes. Targets and cues were presented in six possible locations (Fig 1A) at 8° and 2° of visual angle in x-axis and 5° and 1° in y-axis of visual angle respectively. Leftwards and rightwards target locations were distributed equally, whereas middle target locations were weighted by 50% vs. 25% of upper and 25% of lower locations. The task comprised congruent trials with target stimuli preceded by congruent directional cues (60%, Fig 1B), incongruent trials with target stimuli preceded by incongruent directional cues (15%, Fig 1C), and stimuli without cues (25%, Fig 1D). The total number of stimuli in the task was 500 in each measurement. Targets were presented for 500ms and cues for 200ms. The intertrial interval was varied in the range of 80020133500ms with average of 2200ms. Time interval between cue and target varied between 200 and 700ms with an average of 450ms. The participants were instructed to direct their attention and gaze from fixation point to targets only if they were preceded by a cue. The task lasted about 35 minutes. One week prior to the first experimental day, participants were extensively trained on the experimental task, in order to avoid the influence of a learning process on the number of errors.

### Eye Tracking data acquisition and analysis

Eye position was monitored with Smart Eye Pro (Smart Eye AB, Göteborg, Sweden). This eye tracking system uses the reflections of infrared flashes on the cornea to find the centre of eyes and calculate gaze direction. It has a 60 Hz sampling frequency, a headtracking range ±65° and eye tracking range ±20° of visual angle and a tracking accuracy of 0.5°. The analysis of eye-tracking data was done using the Python language-based program, written for the purpose of the study. Saccades were detected using velocity and distance criteria, i.e. the movement was classified as a saccade only when its velocity reached 35°/s and its distance was at least 2° of visual angle. Oculomotor reaction time was calculated as a difference between target appearance and the beginning of a saccade.

**Fig 2:**
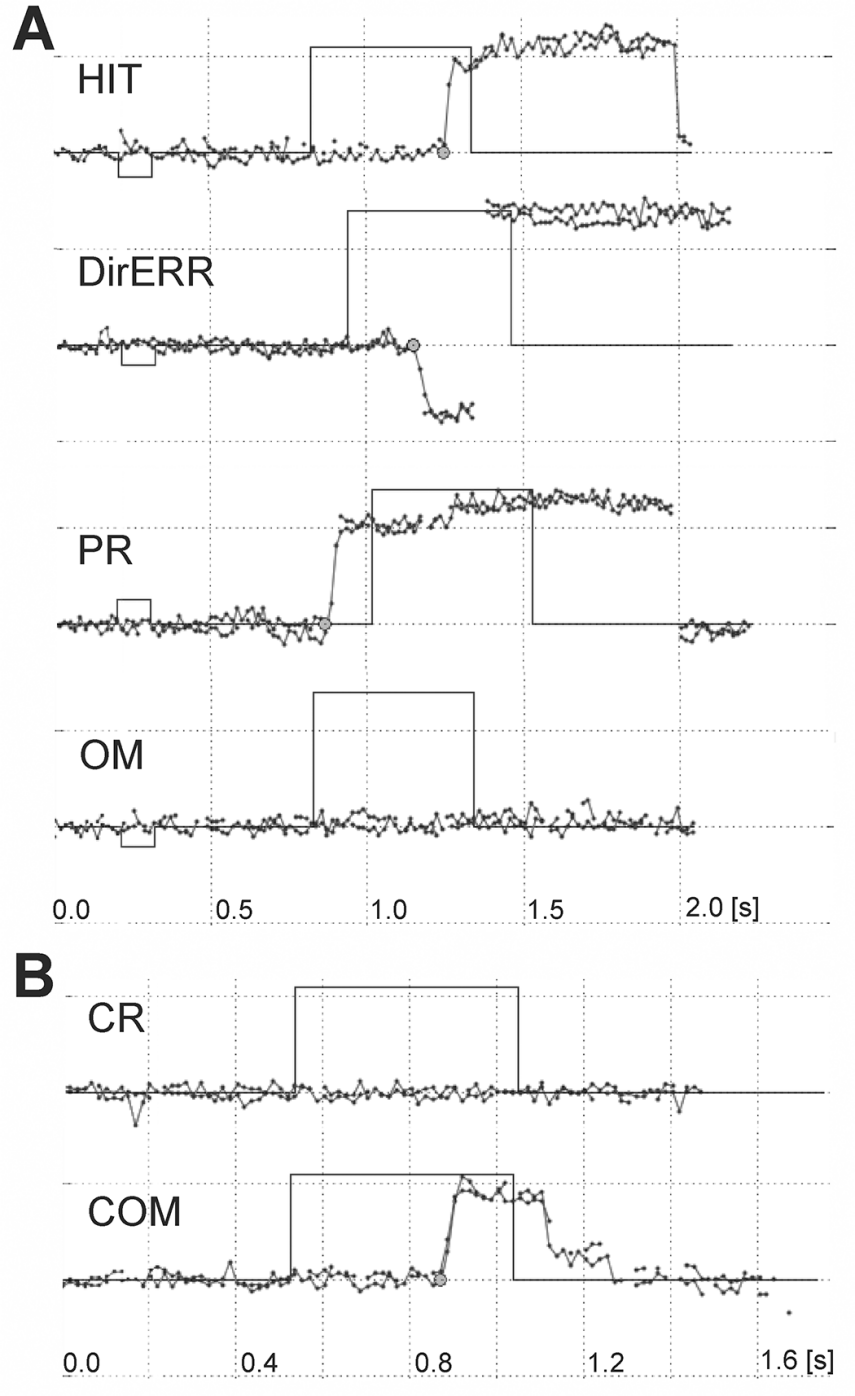
Representative reactions in saccadic task for rightward centered stimuli. Reaction types for (A) stimuli preceded by a cue and for (B) stimuli without a cue. HIT = correct reaction, DirERR = direction error, PR = premature reaction, OM = omission, CR = correct rejection, COM = commision.

Subjects were instructed to react only to targets preceded by a cue. Saccadic reactions were classified according to following criteria. The reactions to a target preceded by a cue (either congruent or incongruent) were recognized as correct (HIT) if the direction of the eye movement was consistent with the target position and if the reaction time was at least 80ms. Saccades executed earlier than 80ms after the stimulus onset (i.e. anticipatory saccades; Fischer et al., 1995) and those before target appearance, regardless of the direction, were labelled premature reactions (PR). A lack of reaction during the stimulus presentation was considered as an omission error (OM). If the direction of the first saccade was opposite to the target location, the reaction was classified as a direction error (DirERR). In trials without a cue, the lack of any saccade was considered a correct rejection (CR). Reaction to the target not preceded by a cue was labelled as an error of commission (COM). Examples of reactions are depicted on Fig 2. Recordings in which the eye tracking signal was poor and classification was not possible were discarded from further analyses. This resulted in an average loss of 6.4% (std.err. 1.3%) of trials.

## Results

The mean length of sleep during the RW condition (8h 9min ± 37min) was significantly longer (*t*_(23)_ = 15.66, *p* < 0.001) than the average length of sleep during the SD condition (5h 41min ± 29min). On average, participants in the SD condition restricted their sleep time by 30% (± 8%) comparing to RW condition. The CHICa score differed significantly between the two conditions (RW: 9.0 ± 8.4, SD: 38.1 ± 16.0, *t*_(23)_ = 8.62, *p* < 0.001).

**Fig 3:**
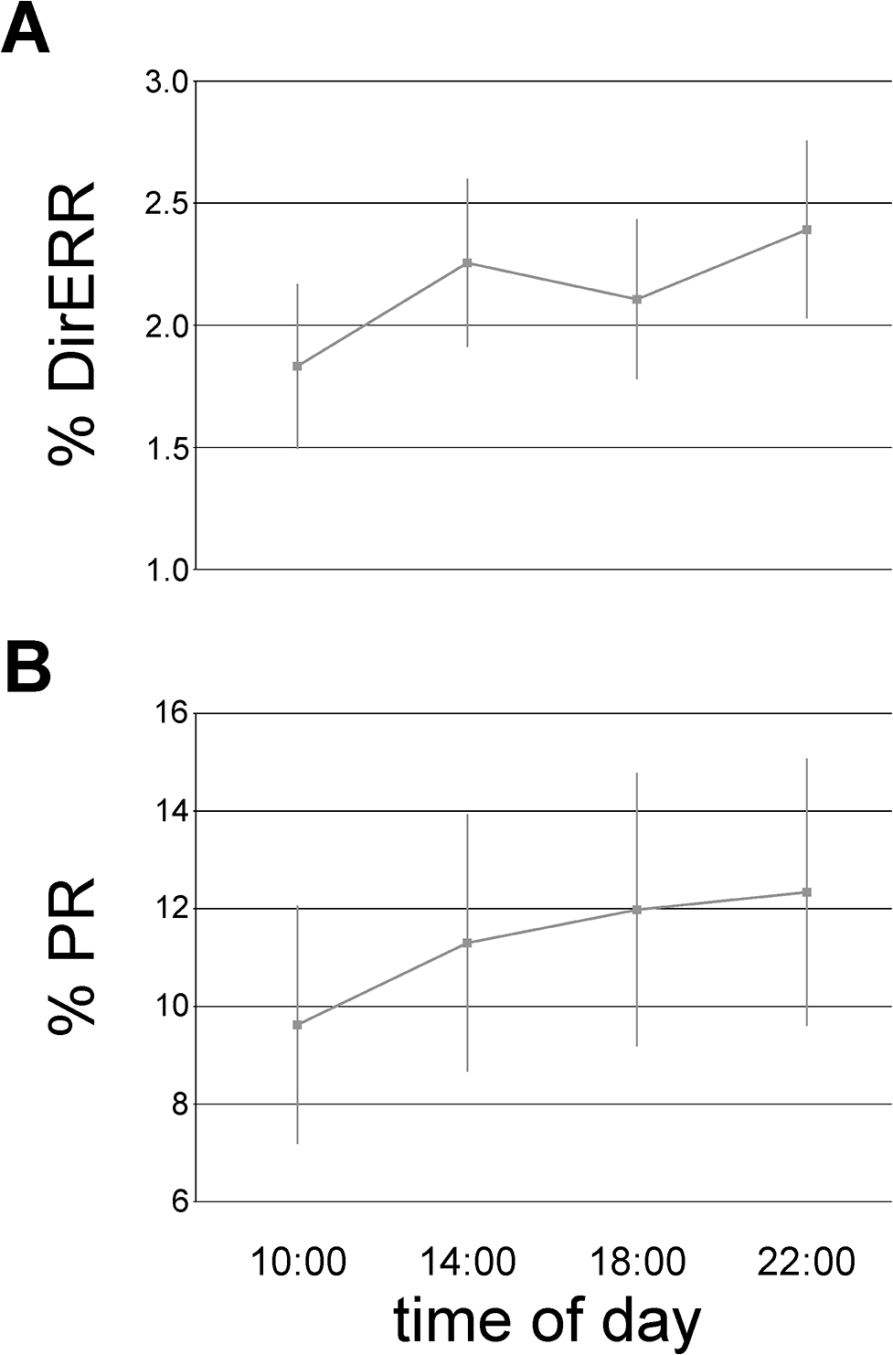
Significant time of day variations for two types of reaction: (A) DirERR; (B) PR. DirERR = direction error, PR = premature reaction.

Overall saccadic task performance measurements are depicted in Table 1. The number of each type of reactions was analysed as a proportion of the overall number of classified reactions. An analysis of the variance (two-way ANOVA) with sleep condition and time of day (TOD) as factors was performed separately for each type of reaction. The analysis showed that the number of DirERRs changes significantly with TOD (*F*_(3,69)_ = 3.51, *p* = 0.02; Fig 3A). A post-hoc LSD test showed that this kind of error was committed significantly more frequently in the first than in the second (*p* = 0.02) and the fourth session (*p* = 0.003). Considering that the abundance of DirERRs were committed on incongruent trials, additional analysis was conducted solely on these trials. The results presented similar TOD variations (F_(3,69)_ = 3.32, *p* = 0.025) to those obtained for both types of trials. The proportion of PR in the number of all trials increased significantly depending on the TOD (*F*_(3, 69)_ = 5.36, *p* = 0.002; Fig 3B). A post-hoc test showed significant difference between the morning session and other times of day: 14:00 (*p* = 0.02), 18:00 (*p* = 0.002), and 22:00 (*p* = 0.0004).

**Table 1:** Performance in saccadic task.

Type of trial	Type of reaction	Percentage (std. err.)	Reaction time (std. err.)
**Targets preceded by a cue**	Correct reactions	83.1% (2.9%)	C: 287ms (7ms); I: 340ms (4ms) *
	Direction errors	2.1% (0.3%)	177 ms (3ms) *
	Premature reactions	11.4% (2.6%)	44 ms (6ms) before target appearance *
	Omissions	1.7% (0.4%)	-
**Targets without a cue**	Correct rejections	88.4% (1.9%)	-
	Commissions	11.6% (1.9%)	403 ms (13ms) *

Note: * indicate significant difference between reaction times in comparison to HIT in congruent trials (p < 0.001); C = congruent, I = incongruent.

**Fig 4:**
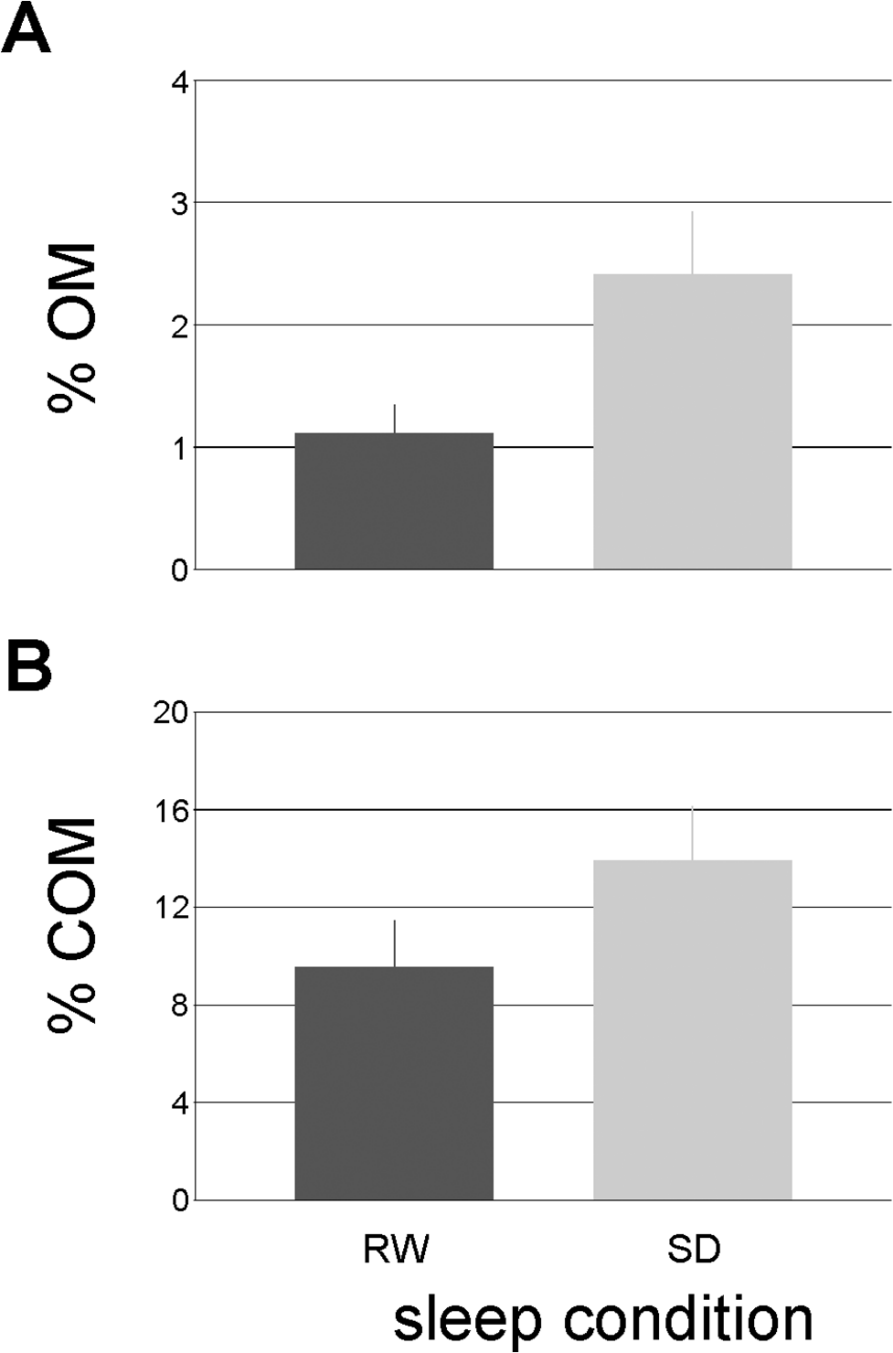
Significant changes between two sleep conditions for two types of reaction: (A) OM; (B) COM. OM = omission, COM = commision.

The COM and OM showed no significant variation across different times of day. The ANOVA revealed a significant difference in the number of these errors between RW and SD conditions. The ratio of OM was significantly higher in the SD condition (*F*_(1,23)_ = 13.61, *p* = 0.001; Fig 4A) as was the proportion of COM (*F*_(1,23)_ = 5.86, *p* = 0.02; Fig 4B). There was no significant interaction of TOD and sleep conditions for any type of reaction.

Changes in subjective sleepiness measured by KSS before each session showed significant variance between RW and SD condition (*F*_(1,23)_ = 68.62, *p* < 0.001) and between the times of the day (*F*_(3,69)_ = 8.21, *p* < 0.001). There was no significant difference between average reaction times, neither between the two sleep conditions, nor between times of the day. Mean latency for all reaction are presented in Table 1.

## Discussion

Following a basic distinction of erroneous responses, errors can be caused both by failures in response execution, when the selected response program is correct but its implementation goes sideways, and failures in response selection, when a wrong, not consistent with the task demands, response program is executed [19]. In this study, we were able to distinguish four types of errors lying beneath those categories. Failures in response execution took place when participants reacted to the cued target before its appearance (PR) or when they made a saccade to the opposite direction of the target location (DirERR). Accordingly, failures in response selection were observed either when participants shifted attention to uncued targets, while, according to the instruction, they were supposed to maintain focus on the fixation point (COM) or when participants did not execute a reaction on a cued target (OM). The different nature of the aforementioned errors suggests them to be associated with separate attentional networks [20]. Namely, the unsuccessful implementation of the action indicates the area of response execution was scanned ineffectively and the reaction was either inadequate or executed to a stimulus not considered a target. It can also be explained in terms of the failure of the orienting network—the mechanism responsible for shifting focus of attention on a potentially relevant area where the stimulus may occur [6]. Respectively, failures in response selection are considered as a result of disruption in alerting network activity [21], crucial to achieving and maintaining readiness to react to incoming stimuli.

The results of our study showed the time of day variations in susceptibility to PR and DirERR. These errors in response execution are related to the cue facilitation mechanism [22] as the cue leads to the orienting of attention and facilitates programming a saccade to the cued location. When the delay between the presentation of the gaze cue and the onset of a target is short, there is an increased tendency to commit DirERR caused by an automatic saccade following the incongruent cues [23]. Facilitation leads not only to DirERR, but also to the anticipatory saccades, in our case the PR, linked with decision-related neural activity [24]. The number of both DirERR and PR was not affected by chronic sleep loss, which is in line with the study of Martella et al. [25], showing that the facilitation effect is invulnerable to sleep deficit. Interestingly, the number of DirERR increases during the post-lunch dip [26] and in the late evening, whereas the number of PR increases constantly during the day. The worsening of performance in the mid-afternoon has already been associated with the decreased efficiency of attention disengagement in the orienting process of attention shifts between different locations [27]. The worsening of performance in the late evening can be explained in terms of the fatigue-related failure in response execution caused by a crash of the orienting attention network. Thus, it can be concluded that the orienting attention sub-system is mostly prone to the circadian and fatigue factors which cause a boost in cue facilitation and prediction mechanisms and the increase of DirERR and PR. These findings are consistent with the results of our previous study [28], which implemented neural measurements under the TOD condition and showed decreasing activity of the orienting attentional system.

The increase in the number of OM under the condition of chronic sleep deficit, similar to the acute condition, can be explained by the lapses of attention related to the wake state instability and microsleeps [2], during which the alerting sub-system of attention is compromised. This explanation is consistent with the evidence that at least subjective alertness and the ability to maintain sustained attention are highly influenced by sleep deprivation [3]. The increased number of COM, i.e. responses to no-go targets, is explained in terms of the diminished ability of the top-down inhibition of dominant automatic response [29]. This is consistent with the findings that response inhibition is particularly sensitive to sleep deprivation and occur even before an increment in the number of OM [30]. It leads to inappropriate response selection regardless of other factors such as the circadian process and the age of sleep-deprived participants [31]. Taken together, the increase in the number of OM and COM is understood in term of changes in the interplay of two attentional processes: bottom-up and top-down, resulting in problems maintaining stable and adequate behavioural reactions [32]. Considering that the errors in response selection can be explained in terms of the failure of the alerting sub-system of attention [6], it can be concluded that this system is highly prone to chronic sleep restriction. It is in agreement with neuroimaging evidence, compensatory activation of the thalamus, the brain region linked with alertness, occurs under the condition of sleep deficit [33].

The variations regarding both the alerting and orienting systems should be considered in relation to the supervisory system of executive control [6]. Indeed, a recent computational account [34] indicated that complex behavioural patterns are a result of the interaction of these three attention systems. The impairment of the executive control system may lead to a failure of top-down processes and as a consequence, the disinhibition of bottom-up processes. On the one hand, the impairment of the executive control network influences the orienting one, as the former is linked with ‘rapid strategic control over attention’, enabling the voluntary switching of attention between cue and target locations [6,12]. Thus, failures of the executive system may lead to changes in the functioning of orienting-related cue facilitation mechanisms, causing premature reactions and direction errors. On the other hand, the impairment of the executive control network also influences the alerting network, leading to increasing stimulus-driven reactions causing an increased number of commissions. Moreover, wake state instability and lapses of attention can be linked with the collapse of all attentional systems mirrored in the increased number of omissions [2]. Taking our results into consideration, the functioning of the executive control network seems to be sensitive to both chronic sleep deficit and different times of day.

## Conclusions

Diverse attentional components and performance indices of sustained attention are differently affected by circadian and homeostatic processes (eg. [7,35]). Attention engagement and related performance are not based on a single cognitive process, but on various processes which can be differently affected by sleep restriction and circadian factors. In this study, we have shown that the time of day variations cause an increase in premature responses and direction errors, whereas chronic sleep deficit causes an increase in omissions and commissions. While the former can be related to the impairment of the orienting attentional system, the latter can be linked to failure of the alerting attentional network. Disruption in the functioning of these two systems can be caused by a decrement in the control ability mediated by the executive system. However, the above conclusions need further investigation, not only on a behavioural but foremost, on a neuronal level.
